# The Tat protein of human immunodeficiency virus-1 enhances hepatitis C virus replication through interferon gamma-inducible protein-10

**DOI:** 10.1186/1471-2172-13-15

**Published:** 2012-04-03

**Authors:** Jing Qu, Qi Zhang, Youxing Li, Weiyong Liu, Lvxiao Chen, Ying Zhu, Jianguo Wu

**Affiliations:** 1 State Key Laboratory of Virology, College of Life Sciences, and Chinese-French Liver Disease Research Institute at Zhongnan Hospital, Wuhan University, Wuhan 430072, P.R. China; 2 Wuhan Institutes of Biotechnology, 666 Gaoxin Road, Wuhan East Lake High Technology Development Zone, Wuhan 430075, P.R. China; 3 State Key Laboratory of Virology and College of Life Sciences, Wuhan University, Wuhan 430072, P.R. China

## Abstract

**Background:**

Co-infection with human immunodeficiency virus-1 (HIV-1) and hepatitis C virus (HCV) is associated with faster progression of liver disease and an increase in HCV persistence. However, the mechanism by which HIV-1 accelerates the progression of HCV liver disease remains unknown.

**Results:**

HIV-1/HCV co-infection is associated with increased expression of interferon gamma-induced protein-10 (IP-10) mRNA in peripheral blood mononuclear cells (PBMCs). HCV RNA levels were higher in PBMCs of patients with HIV-1/HCV co-infection than in patients with HCV mono-infection. HIV-1 Tat and IP-10 activated HCV replication in a time-dependent manner, and HIV-1 Tat induced IP-10 production. In addition, the effect of HIV-1 Tat on HCV replication was blocked by anti-IP-10 monoclonal antibody, demonstrating that the effect of HIV-1 Tat on HCV replication depends on IP-10. Taken together, these results suggest that HIV-1 Tat protein activates HCV replication by upregulating IP-10 production.

**Conclusions:**

HIV-1/HCV co-infection is associated with increased expression of IP-10 mRNA and replication of HCV RNA. Furthermore, both HIV-1 Tat and IP-10 activate HCV replication. HIV-1 Tat activates HCV replication by upregulating IP-10 production. These results expand our understanding of HIV-1 in HCV replication and the mechanism involved in the regulation of HCV replication mediated by HIV-1 during co-infection.

## Background

Hepatitis C virus (HCV) is a major etiological agent of chronic liver disease. An estimated 180 million humans are infected with HCV worldwide. Due to similar routes of transmission, co-infection with HCV and human immunodeficiency virus-1 (HIV-1) is common, with the prevalence of co-infection ranging from 4 to 5 million patients [1]. HCV-related liver diseases have become a major source of morbidity and mortality in HIV-1-infected patients [2]. Once chronic infection is established, patients with HIV-1/HCV co-infection have a higher rate of viral persistence, faster progression, and earlier development of end-stage liver disease, compared to HCV mono-infected patients [3,4]. Infection with HIV-1 is associated with higher HCV viral levels in sera compared to infection with HCV alone [5]. However, the mechanisms that accelerate progression of HCV/HIV-1 co-infected patients are not fully understood.

HIV-1 infection enhances HCV replication, thus changing the course of HCV-related disease in co-infected patients [6,7]. HCV was originally thought to be strictly hepatotropic, while the main cell targets for HIV-1 infection are mononuclear leukocytes bearing CD4 and the chemokine receptors C-C chemokine receptor type 5 (CCR5) and chemokine (C-X-C motif) receptor 4 (CXCR4). However, HCV can also replicate in peripheral blood mononuclear cells (PBMCs), particularly in patients with HIV-1 [8,9]. The effect of HIV-1 on PBMC cultures of HCV mono-infected patients *in vitro *has previously been investigated. The production of HCV post-HIV infection increases by 1 to 2 logs, compared to uninfected controls [10]. Also, HIV-1 facilitates replication of HCV in native human macrophages *in vitro *[11].

The interferon γ-inducible protein 10 (IP-10 or CXCL10) is a chemotactic C-X-C chemokine that attracts activated T-lymphocytes and monocytes [12-14]. IP-10 is produced by a variety of cells, including astrocytes and hepatocytes [15,16]. Increased levels of IP-10 have been detected in the serum and liver of HCV-infected individuals compared to controls [17,18]. Elevated IP-10 is correlated with increased liver damage [19] and HCV viral loads [20], as well as enhanced IP-10 levels in HIV-1 mono-infected patients compared to controls [21]. Increased IP-10 production during HIV-1 infection has been partially attributed to HIV-1 proteins, including HIV-1 accessory protein transactivator of transcription (Tat), in a number of cells such as astrocytes and macrophages [22,23]. Serum IP-10 levels are higher in HIV-1/HCV co-infected patients than in HCV mono-infected patients [24].

HIV-1 Tat is a transactivating protein that contributes to the transactivation of viral and cellular genes [25]. Extracellular Tat, released from virus-infected cells, can enter neighboring infected or uninfected cells and induce its biological effects, including cytokine expression [26,27]. For example, extracellular Tat stimulates IL-10 expression in human monocytes in a time- and dose-dependent manner [28]. Also, Tat upregulates the expression of specific chemokine receptors, such as CCR5 and CXCR4, which are important for HIV-1 infection [29]. In addition to its regulatory role in HIV-1 infection, Tat may activate [30,31] and facilitate the invasion of viruses [32].

IP-10 mRNA levels in PBMCs from HIV-1/HCV co-infected and HCV mono-infected patients showed that HIV-1/HCV co-infection was associated with increased expression of IP-10 mRNA and in the replication of HCV RNA. Furthermore, we used two different infectious HCV models to examine the effects of HIV-1 Tat and IP-10 on HCV replication, which demonstrated that both HIV-1 Tat and IP-10 activate HCV replication. Also, HIV-1 Tat activates HCV replication by upregulating IP-10 production. The mechanism involved in the regulation of HCV replication mediated by HIV-1 during co-infection is discussed.

## Results

### IP-10 mRNA and HCV RNA levels are increased in patients with HIV-1/HCV co-infection compared to HCV mono-infection

**Figure 1:**
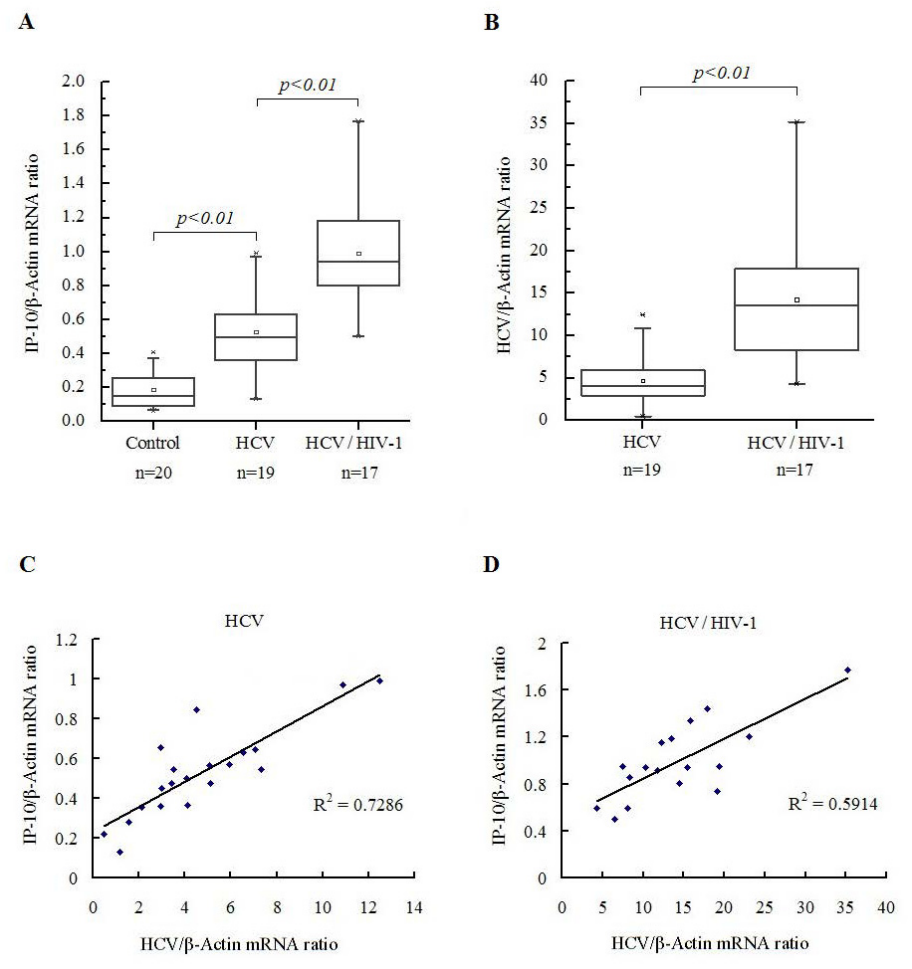
**Analysis of IP-10 mRNA and HCV RNA levels in PBMCs isolated from healthy individuals, HCV mono-infected patients, and HIV-1/HCV co-infected patients**. (A) Transcriptional expression of IP-10 in PBMCs isolated from HCV mono-infected patients, HIV-1/HCV co-infected patients, and healthy individuals. Data were normalized to the amount of GAPDH-specific mRNA as a reference transcript. Numbers below each patient group (e.g. *n *= 24) indicate the number of persons in that group. (B) Replication of HCV RNA in PBMCs isolated from patients with HCV mono-infection and HIV-1/HCV co-infection. Data were normalized to the amount of GAPDH-specific mRNA as a reference transcript. (C) Correlations between the expression of IP-10 mRNA and HCV RNA in PBMCs isolated from patients with HCV mono-infection and HIV-1/HCV co-infection. (D) Correlations between the expression of IP-10 mRNA and HCV RNA in PBMCs isolated from patients with HIV-1/HCV co-infection. Two groups of patients were analyzed for IP-10 mRNA and HCV RNA expression. Data were subjected to linear regression analysis. Correlation coefficients are indicated.

We evaluated the IP-10 mRNA levels in PBMCs isolated from healthy individuals and HCV mono-infected and HIV-1/HCV co-infected patients. IP-10 mRNA levels were higher in all infected patients compared to healthy control subjects, and higher in patients with HIV-1/HCV co-infection than in those with HCV mono-infection (Figure 1A). Increases in serum HCV viral loads in the HIV-1/HCV co-infected group were more common than in the HCV mono-infected group (median levels: 6.1 × 105 vs. 1.4 × 106 copies/ml, respectively) (Table 1). HCV can also replicate in PBMCs [8,9], so we compared the levels of HCV RNA in PBMCs of HCV mono-infected and HIV-1/HCV co-infected patients. HCV RNA levels were more than three-fold higher in HIV-1/HCV co-infected patients than in HCV mono-infected patients (Figure 1B). These findings are consistent with other studies, demonstrating that HCV RNA levels and viral loads are increased in co-infected patients [33,34].

**Table 1:** Summary of patient characteristics

	HCV	HCV/HIV
	Mono-infected	Co-infected
Characteristic	(n = 20)	(n = 17)
Gender		
Female	8	0
Male	12	17
Age (years)	37 (26-60)	34 (28-38)
ALT level (IU/L)	40.6 (14-118)	9 (2-25)
AST level (IU/L)	34.8 (17-116)	52 (29-99)
CD4 count (cells/ul)	NT	256 (106-474)
CD8 count (cells/ul)	NT	989 (357-1600)
Serum HCV RNA (copies/ml)	6.1 × 10^5 ^(10^3^-1.2 × 10^7^)	1.4 × 10^6 ^(10^3^-6.8 × 10^7^)
HCV genotype		
1	7	8
2	5	4
3	5	3
Unknown	3	2

Data are median (range) values, unless otherwise indicated. ALT, alanine aminotransferase; AST, aspartate aminotransferase; NT, not tested

We examined correlations between IP-10 mRNA levels and HCV RNA levels in both types of patient. Increased IP-10 levels were positively correlated with HCV RNA levels in both groups (Figure 1C, D).

### IP-10 increases HCV replication in Huh7.5.1 cells

Chemokines, which are chemotactic cytokines, play an important role in the pathogenesis of chronic HCV and are useful for the clinical management of patients with hepatitis C [35]. It is also known that chemokine CXCL-8 (interleukin-8) may enhance HCV replication [36].

**Figure 2:**
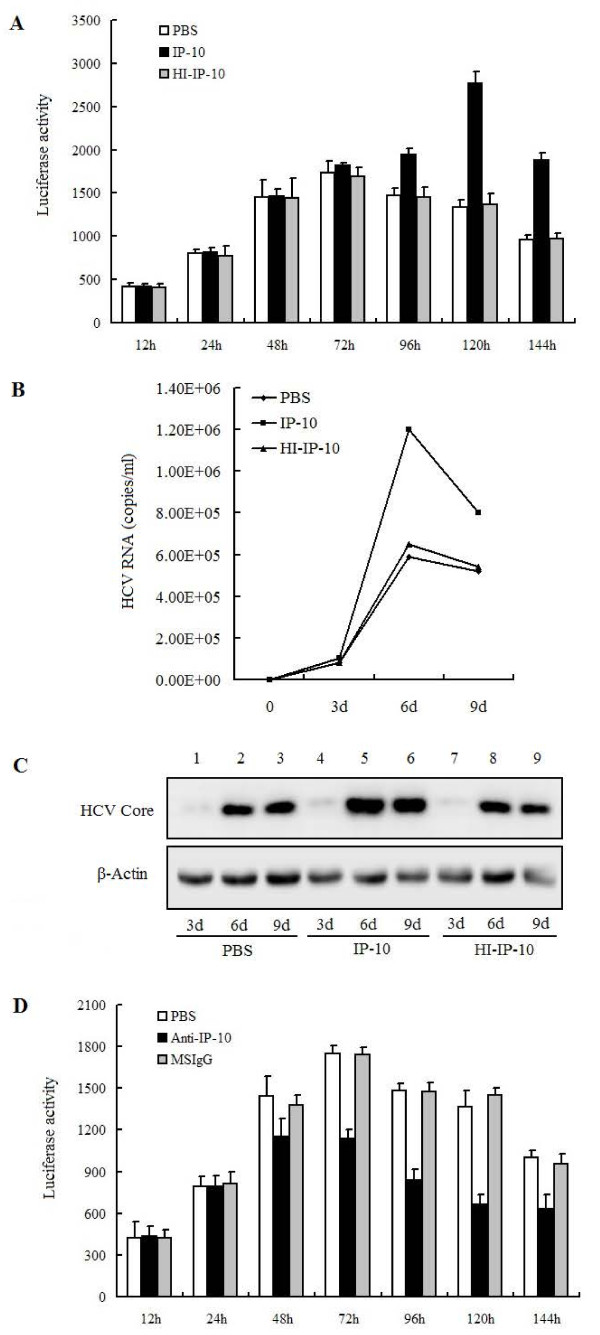
**Determination of the roles of IP-10 protein in the activation of HCV replication**. (A) Huh7.5.1 cells were infected with FL-J6/JFH-5'C19Rluc2Aubi virus for 24 h, and then incubated with PBS, IP-10 (1 μg/ml), or heat-inactivated IP-10 (HI-IP-10) (1 μg/ml) in 24-well plates for different times, as indicated. Treated cells were harvested and analyzed for luciferase activity. (B) Huh7.5.1 cells were infected with JFH1 virus, and then incubated with PBS, IP-10, or HI-IP-10 for 9 days. Culture supernatants were harvested and HCV copy numbers were determined by real-time PCR (RT-PCR). (C) Huh7.5.1 cells were infected with JFH1 virus, and then incubated with PBS, IP-10, or HI-IP-10 for 9 days. Cells were harvested and analyzed for HCV core protein expression by Western blot. β-Actin expression was measured as a loading control. (D) Huh7.5.1 cells were infected with FL-J6/JFH-5'C19Rluc2Aubi virus and then incubated with PBS, 1 μg/ml MSIgG, or 1 μg/ml anti-IP-10 neutralizing antibody for 144 h. Cells were harvested at different times as indicated and luciferase activity was assayed.

We next examined the effects of IP-10 on HCV replication. Huh7.5.1 cells were infected with FL-J6/JFH-5'C19Rluc2Aubi and incubated with phosphate buffer solution (PBS), IP-10, or heat-inactivated IP-10 (HI-IP-10) proteins. Treated cells were harvested and examined for luciferase activity, which increased over time (Figure 2A). However, luciferase activities increased from 96 to 140 h post-treatment by exposure to IP-10, but not PBS or HI-IP-10 (Figure 2A).

To corroborate these results, Huh7.5.1 cells were infected with JFH1 virus, a full cell culture-infectious genotype 2a HCV isolate [37], and then incubated with PBS, IP-10, or HI-IP-10, respectively, for 3, 6, and 9 days. The HCV replication levels in the cellular supernatants were measured, which showed that HCV RNA copies increased over time in the presence of BSA, IP-10, and HI-IP-10, respectively. However, HCV RNA levels were elevated at 3 days and peaked at 6 days post-treatment (Figure 2B). The intracellular levels of HCV core protein was determined by Western blot analyses, demonstrating that IP-10 activates HCV core protein expression from 6 to 9 days post-treatment (Figure 2C). All of these results suggest that IP-10 can activate HCV replication.

Having demonstrated that the addition of IP-10 stimulates HCV replication, we next wanted to determine whether endogenous IP-10 is also involved in HCV replication. We examined the effects of anti-IP-10 antibodies on HCV replication. Huh7.5.1 cells were infected with FL-J6/JFH-5'C19Rluc2Aubi and incubated with IP-10 neutralizing antibody, PBS, or MSIgG (as a control). IP-10 neutralizing antibody inhibited HCV replication (Figure 2D). In contrast, PBS and MSIgG did not affect HCV replication. Therefore, endogenous IP-10 is involved in the activation of HCV replication.

### HIV-1 tat protein induces IP-10 production

**Figure 3:**
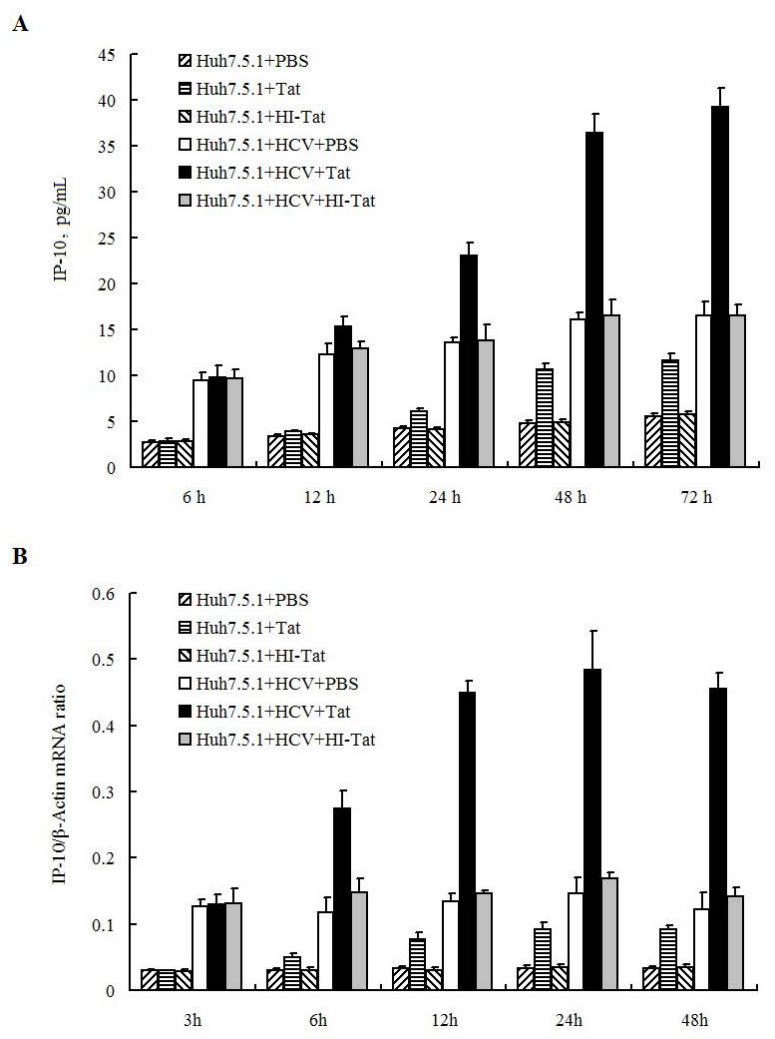
**Analysis of the role of HIV-1 Tat protein in the regulation of IP-10 expression**. (A) Huh7.5.1 cells were infected with or without JFH1 virus and then incubated with PBS, 1 μg/ml Tat, or 1 μg/ml heat-inactivated Tat (HI-Tat) for different times, as indicated. Cell culture supernatants were collected and IP-10 levels were quantified by ELISA. (B) Huh7.5.1 cells were infected with or without JFH1 virus and then incubated with PBS, 1 μg/ml Tat, or 1 μg/ml heat-inactivated Tat (HI-Tat) for different times, as indicated. Cells were collected and the levels of IP-10 mRNA were quantified by real-time PCR (RT-PCR).

HIV Tat is known to induce IP-10 production in human astrocytes [22]. Huh7.5.1 cells were infected with or without JFH1 and then treated with PBS, Tat, or heat-inactivated Tat (HI-Tat), respectively. IP-10 protein levels were detected by ELISA at different times post-treatment. IP-10 protein levels were significantly higher in HCV-infected cells than in uninfected cells (Figure 3A), indicating that HCV activates IP-10 expression. HIV-1 Tat protein induced IP-10 protein production in a time-dependent manner in both HCV-infected and uninfected cells (Figure 3A), demonstrating that Tat can activate IP-10 protein expression.

We next measured the IP-10 mRNA levels in Huh7.5.1 cells infected with or without JFH1 and treated with PBS, Tat, or HI-Tat, respectively. IP-10 mRNA levels were higher in HCV-infected cells than in uninfected cells (Figure 3B), indicating that HCV activates IP-10 gene expression. HIV-1 Tat protein induced IP-10 mRNA expression in a time-dependent manner in both HCV-infected and uninfected cells (Figure 3B), suggesting that Tat can activate IP-10 gene expression at the transcriptional level.

### HIV-1 Tat protein stimulates HCV replication

**Figure 4:**
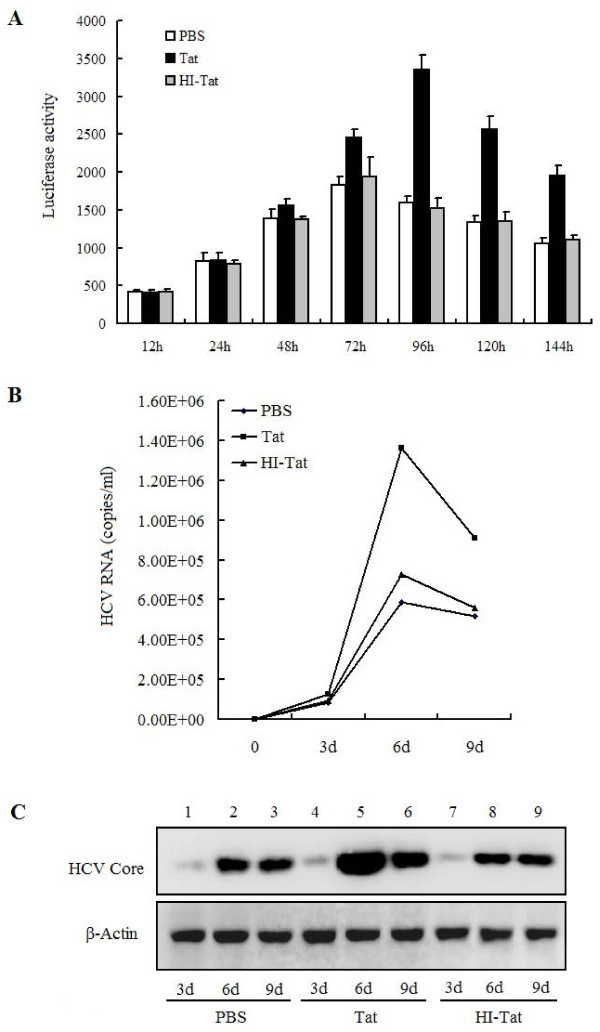
**Determination of the role of HIV-1 Tat protein in the activation of HCV replication**. (A) Huh7.5.1 cells were infected with FL-J6/JFH-5'C19Rluc2Aubi virus and then incubated with PBS, 1 μg/ml Tat, or 1 μg/ml HI-Tat in 24-well plates for different times, as indicated. Cells were harvested and analyzed for luciferase activity. (B) Huh7.5.1 cells were infected with JFH1 virus and then incubated with PBS, Tat, or HI-Tat for 9 days. Cell culture supernatants were harvested and HCV copy numbers were determined by real-time PCR (RT-PCR). (C) Huh7.5.1 cells were infected with JFH1 virus and then incubated with PBS, Tat, or HI-Tat for 9 days. Cells were harvested and HCV core protein levels were detected by Western blot analyses. β-actin level was determined as a loading control.

Our previous study showed that Tat up-regulated HCV replication using co-transfection assay [38]. In this study, we assessed the effects of HIV-1 Tat protein on HCV replication in Huh7.5.1 cells infected with FL-J6/JFH-5'C19Rluc2Aubi and treated with PBS, Tat, or HI-Tat, respectively. The roles of Tat in the regulation of HCV replication were determined by measuring luciferase activities, which showed that Tat protein, but not PBS or HI-Tat, activated HCV replication 72 h post-treatment. The HCV replication levels activated by Tat peaked 96 h post-treatment and then declined (Figure 4A).

We next evaluated the role of Tat in the regulation of HCV replication using a second system. Huh7.5.1 cells infected with JFH1 virus were incubated with PBS, Tat, or HI-Tat, respectively. HCV RNA copies were measured in the cell culture supernatant, which showed that HCV RNA copies increased over time until 6 days post-treatment (Figure 4B). The HCV RNA levels were elevated in the presence of Tat, but not in the presence of PBS or HI-Tat (Figure 4B), indicating that HIV-1 Tat protein stimulates HCV RNA replication. The HCV core protein levels were also measured by Western blot analyses, indicating that core protein levels increased over time until 6 days post-treatment (Figure 4C). In addition, HCV core protein levels were elevated in the presence of Tat, but not in the presence of PBS or HI-Tat (Figure 4C), suggesting that HIV-1 Tat protein can activate HCV core protein expression.

### Anti-IP-10 antibodies block the activation of HCV replication regulated by HIV-1 Tat

**Figure 5:**
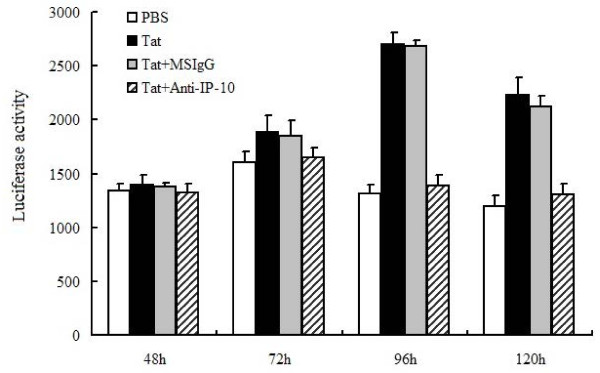
**Role of endogenous IP-10 in the activation of HCV replication regulated by HIV-1 Tat protein determined by antibody depletion**. Huh7.5.1 cells were infected with FL-J6/JFH-5'C19Rluc2Aubi virus and then incubated with PBS, Tat, Tat plus MSIgG, and Tat plus anti-IP-10 neutralizing antibody, respectively, in 24-well plates for 120 h. Cells were then harvested at different times (as indicated) and luciferase activity was assayed.

Both HIV-1 Tat and IP-10 enhance HCV replication, and HIV-1 Tat can increase IP-10 expression levels in hepatocytes. However, whether HIV-1 Tat activation of HCV replication is dependent on IP-10 remains unknown. Huh7.5.1 cells infected with FL-J6/JFH-5'C19Rluc2AUbi were incubated with PBS, Tat, Tat plus MSIgG, or Tat plus anti-IP-10 neutralizing antibodies. HIV-1 Tat protein activated HCV replication in Huh7.5.1 cells treated with PBS or MSIgG, but not in cells treated with anti-IP-10 neutralizing antibodies (Figure 5). Taken together, these results suggest that IP-10 enhances HCV replication and HIV-1 Tat activates HCV replication by regulating IP-10 during HIV-1/HCV co-infection.

## Discussion

Co-infection of HCV with HIV-1 is prevalent and causes increased HCV viral loads, as well as increased morbidity in co-infected individuals [39,40]. The precise molecular mechanism by which HIV-1 increases HCV persistence and accelerates liver fibrosis is unknown. Chemokines play an important role in the pathogenesis of both HCV and HIV-1 infections. Also, HIV-1 Tat protein is known to activate HCV replication by upregulating IP-10 production.

Elevated IP-10 levels have been detected in HCV-infected patients compared to healthy controls [17,18]. This association is supported by the positive correlation between IP-10 levels and HCV viral load, as well as the significant decrease in IP-10 levels after clearance of HCV infection [17]. Elevated IP-10 mRNA levels were detected in HCV mono-infected patients compared to healthy controls. Also, increased IP-10 mRNA levels were associated with increased HCV RNA levels in HCV-infected patients. This indicates that IP-10 plays an important role in the natural pathogenesis of HCV-induced liver damage. Furthermore, IP-10 promotes HIV-1 replication in monocyte-derived macrophages and peripheral blood lymphocytes [41]. However, the effect of IP-10 on HCV replication is not well understood. IP-10 upregulates HCV replication in hepatocytes in a time-dependent manner. Treatment with an IP-10-specific monoclonal antibody reduces HCV replication in Huh7.5.1 cells, demonstrating that endogenous IP-10 stimulates HCV replication.

Previous studies have reported that IP-10 levels are elevated in HIV-1 mono-infected patients compared to healthy control subjects [42], and that IP-10 stimulates HIV-1 replication [41]. Significantly higher serum IP-10 levels have been reported in HIV-1/HCV co-infected patients than in mono-infected and uninfected control groups [24]. This suggests that IP-10 plays an important role in the pathogenesis of HIV-1/HCV co-infection. However, no reports are available on the role of IP-10 in the regulation of HIV-1/HCV co-infection. Initially, we measured the IP-10 mRNA levels in PBMCs from a healthy, HCV mono-infected, and HIV-1/HCV co-infected group. HIV-1/HCV co-infection was associated with significantly increased IP-10 mRNA expression in PBMCs compared to HCV mono-infection, suggesting that HIV-1 enhances IP-10 expression in HCV/HIV-1 co-infected patients.

The transactivating effects of Tat are not limited to HIV-1-infected cells because extracellular Tat is secreted into the plasma of HIV-1-infected patients where it can exert its effects on uninfected cells [43]. Extracellular Tat non-specifically binds to cell membranes and is internalized, which may induce intracellular signals that ultimately change cellular gene expression. For example, Tat upregulates the expression of a number of cytokines (TNF-α, IL-10) [44,45] and chemokines (IL-8, IP-10) [22]. HIV-1 Tat also induces IP-10 in astrocytes [22,46]. Our results corroborate these findings because IP-10 expression was increased in Huh7.5.1 cells in the presence of HIV-1 Tat. Also, HCV infection upregulates IP-10 expression in Huh7.5.1 cells. This is consistent with *in vivo *findings that demonstrated that IP-10 mRNA levels were higher in HCV-infected patients than in healthy controls. We found that Tat activates IP-10 expression and HCV replication, and that IP-10 upregulates HCV replication in two infectious HCV models. Treatment with an anti-IP-10 monoclonal antibody reduced Tat-activated HCV replication, demonstrating that Tat enhancement of HCV replication is dependent on IP-10. Tat has been implicated in the activation of cytokine transforming growth factor (TGF)-β in HIV-1-infected cells and in the stimulation of JC virus (JCV) gene transcription and DNA replication in oligodendroglia, the primary central nervous system cell type infected by JCV in progressive multifocal leukoencephalopathy [47]. Tat also activates Kaposi sarcoma-associated herpesvirus (KSHV) lytic cycle replication from latency, in part by modulating janus kinase/signal transducer and activator of transcription (JAK/STAT) pathways, and promotes KS progression [48]. HIV-1 transactivator protein Tat, produced by HIV-1-infected cells, is secreted into the extracellular matrix where it activates a complex cascade of signaling pathways and alters the intrahepatic chemokine environment, particularly chemokine IP-10. This contributes to the activation of HCV replication and accelerates liver fibrosis observed in patients co-infected with HIV-1 and HCV. The increased level of IP-10 induction in PBMCs from HIV-1-infected individuals may allow for the establishment of more aggressive HCV infection. The downregulation of anti-HCV responses allow for increased HCV replication and higher HCV viral loads. Tat activation of IP-10 may promote HIV-1 immunosuppression and stimulate HCV gene transcription. Elevated IP-10 levels in HCV mono-infected patients are correlated with increased liver disease. The increased IP-10 levels in HCV/HIV-coinfected patients may explain the accelerated progression to liver disease in these individuals. IP-10 expression is also associated with immunological treatment failure following HAART [21]. Therefore, reducing IP-10 expression and function may enhance the effectiveness of current anti-retroviral treatments.

## Conclusions

HIV-1/HCV co-infection is associated with a significant increase in the expression of IP-10 mRNA in PBMCs. HCV RNA levels were higher in PBMCs of patients with HIV-1/HCV co-infection than in those with HCV mono-infection. HIV-1 Tat or IP-10 activates HCV replication in a time-dependent manner. Also, HIV-1 Tat induces IP-10 production. In addition, the effect of HIV-1 Tat on HCV replication was blocked by anti-IP-10 monoclonal antibody, demonstrating that the effect of HIV-1 Tat on HCV replication depends on IP-10. Taken together, these results suggest that HIV-1 Tat protein activates HCV replication by upregulating the production of the chemokine IP-10. Thus, IP-10 may be a potential therapeutic target for the treatment of patients with HIV-1/HCV co-infection. Further work is necessary to determine the intermediary pathways responsible for the effects on HCV replication. Our study increases the understanding of the mechanisms involved in the co-infection of two deadly viruses, HIV-1 and HCV.

## Methods

### Patient samples

PBMCs from 20 HCV mono-infected and 17 HIV-1/HCV co-infected patients were obtained from local AIDS clinics and hospitals. Patients were excluded if they were positive for hepatitis B virus (HBV) surface antigens or had any of the following: renal failure, hepatocellular carcinoma, decompensated liver disease, or any cause of liver disease other than HCV infection. The demographic information on these patients is summarized in Table 1. PBMCs isolated from the blood of 24 age-matched healthy persons collected at a local blood donation center were used as controls. Informed consent was obtained from all patients. Collection of blood samples for research was approved by the Institutional Review Board of the College of Life Sciences, Wuhan University, China, in accordance with the guidelines for the protection of human subjects.

### Reagents

Recombinant HIV-1 Tat (ab83353) was purchased from Abcam. Recombinant human IP-10 (266-IP-010) and mouse immunoglobulin G (MSIgG) were purchased from R&D Systems. Antibody against the HCV core protein (sc-52804) and IP-10 (sc-28877) were purchased from Santa Cruz, and antibody against β-actin (cw-0096A) was purchased from CWBio.

### Cells and virus stocks

Huh7.5.1 cells were cultured in Dulbecco's modified eagle medium (DMEM) (Gibco BRL, USA) supplemented with 10% fetal calf serum (Gibco BRL, USA), 100 U/ml penicillin, and 100 μg/ml streptomycin sulfate. The cells were maintained at 37°C in a 5% CO2 incubator.

The genotype 2a HCV virus, JFH1, was prepared and used to infect Huh7.5.1 cells, as previously reported [37]. FL-J6/JFH-5'C19Rluc2AUbi plasmid was kindly provided by Dr. Charles M. Rice, from Rockefeller University and FL-J6/JFH-5'C19Rluc2AUbi virus was prepared as described previously [49]. Virus titration was measured using a commercial kit (HCV RNA qPCR Diagnostic Kit, KHB).

### Luciferase reporter gene assay

To harvest samples for luciferase assays, cells were washed once with phosphate buffered solution (PBS), and then 100 μl lysis buffer (Promega) was added to each well of a 24-well plate, and a 50 μl sample was mixed with luciferase assay substrate (Promega). Luciferase activity was typically measured for 10 s using a luminometer (Turner Designs TD-20/20). Assays were performed in triplicate and are expressed as the mean ± standard deviation (SD) of luciferase activity.

### Real-time RT-PCR

Total RNA was extracted from PBMCs or cells using Trizol reagent (Invitrogen), according to the manufacturer's protocol. DNA was removed from the sample by on-column DNase I treatment. RNA was used as a template to synthesize cDNA using random primers and MMLV-RT (Promega) at 42°C for 60 min, followed by denaturation for 10 min at 70°C. Real-time reverse transcriptase polymerase chain reaction (RT-PCR) was performed using SYBR Green PCR master mix (Roche) in LightCycler 480 (Roche). After an initial incubation at 95°C for 5 min, reaction mixtures were subjected to 40 cycles of amplification under the following conditions: 94°C for 15 s, 55°C for 15 s, and 72°C for 20 s. To examine the quality of detection primers, the fluorescence was measured at this step, followed by a final melting curve step from 50°C to 95°C. Primers for HCV detection were 5'-TCGTATGATACCCGATGCT-3' (forward) and 5'-GTTTGACCCTTGCTGTTGA-3' (reverse). Primers for IP10 detection were 5'-TCTAGAACCGTACGCTGTACCTGC-3' (forward) and 5'-CTGGTTTTAAGGAGATCT-3' (reverse). Primers for internal reference GAPDH detection were 5'-AAGGCTGTGGGCAAGG-3' (forward) and 5'-TGGAGGAGTGGGTGTCG-3' (reverse). Each sample was run in triplicate; threshold cycles (CT) were averaged and normalized to endogenous GAPDH. The relative amount of amplified product was calculated using the comparative CT method (Roche).

### Western blotting and ELISA

Protein was resolved on a 12% sodium dodecyl sulfate-polyacrylamide gel electrophoresis (SDS-PAGE) gel and transferred to nitrocellulose membranes (minipore). Membranes were blocked with PBS containing 5% fat-free milk powder and 0.1% Tween 20 for 1 h, blotted with the appropriate primary antibodies for 2 h, incubated with HRP-conjugated secondary antibody (GE) for 1 h, washed, incubated with HRP substrate luminol reagent (Millipore), and analyzed using a Luminescent Image Analyzer (Fujifilm LAS-4000). IP-10 protein levels in supernatants were measured using a commercially available enzyme-linked immunosorbent assay (ELISA) kit (BD Biosciences).

### Statistical Analyses

Data that were Gaussian distributed were analyzed by parametric *t*-tests for independent samples or by analysis of variance (ANOVA). Correlation coefficients were calculated by linear regression analysis.

## Abbreviations

HIV-1: Human immunodeficiency virus-1; HCV: Hepatitis C virus replication; HBV: Hepatitis B virus; JCV: JC virus; KSHV: Kaposi sarcoma-associated herpesvirus; IP-10 or CXCL10: Interferon gamma-inducible protein-10; Tat: Transactivator of transcription; CCR5: C-C chemokine receptor type 5; CXCR4: Chemokine (C-X-C motif) receptor 4; CXCL8: Interleukin-8 (IL-8); TGF: Transforming growth factor; AIDS: Acquired immure deficiency syndrome; HAART: Highly active antiretroviral therapy; PBMC: Peripheral blood mononuclear cell; MSIgG: Mouse immunoglobulin G; RT-PCR: Real-time reverse transcriptase polymerase chain reaction; PBS: Phosphate buffer solution.

## Authors' contributions

JQ designed and performed the experiments, and drafted the manuscript. QZ performed some of the infection assays. YL, WL and LC participated in recruitment of patient samples. JW and YZ designed and coordinated the study. All authors read and approved the final manuscript.
